# Structural organization of the gynoecium and pollen tube path in Himalayan sea buckthorn, *Hippophae rhamnoides* (Elaeagnaceae)

**DOI:** 10.1093/aobpla/plt015

**Published:** 2013-02-27

**Authors:** Yash Mangla, Rajesh Tandon, Shailendra Goel, S. N. Raina

**Affiliations:** 1 Department of Botany, University of Delhi, Delhi 110 007, India; 2 Amity Institute of Biotechnology, Amity University, Sector 125, Noida 210 303, Uttar Pradesh, India

**Keywords:** Angiospermy, conduplication, dry stigma, progamic phase, ventral pore, ventral slit

## Abstract

Closure of carpels or angiospermy, a key developmental innovation, has been accomplished through different ontogenic routes among the flowering plants. The mechanism of angiospermy produces structural novelties in the gynoecium, which in turn affects the progamic phase. In this paper, we present the structural details of the gynoecium and functional attributes of the progamic phase of *Hippophae rhamnoides*, a dioecious species of Elaeagnaceae. The gynoecium is unicarpellate, and the carpel is dorsiventrally symmetric and conduplicate. The pollen tube path comprises a prominent, ventrally localized dry and non-papillate stigma, a pseudostyle and a dorsally protruded superior ovary. The pollen tube path in the stigmatic region is subdermal, and from the pseudostyle onwards, it resides over the epidermis of conduplicated margins. The epidermal cells along this region are secretory but produce sparse extracellular matrix. The tube approaches the solitary ovule through a tiny conduit in the carpel, the ventral pore. The duration of the entire progamic phase is ∼72 h. The observed mean pollen tube length from stigma to ovule was 908.13 ± 180 µm and the mean tube growth rate was 18.75 µm h^−1^. The study demonstrates that sea buckthorn, a core eudicot, has a simple gynoecium with a pollen tube pathway that incorporates elements of both completely externalized and internalized compitum.

## Abstract (precis)

Gynoecium of *Hippophae rhamnoides* (Elaeagnaceae) is comprised of a single carpel and develops by enfolding of the carpel margins. The enfolding results in a vertical slit over the ventral surface of ovary. The pollen tube path is initially sub-stigmatic and is subsequently along the epidermal surface of slit. The tube accesses the solitary ovule through a pore positioned in the slit. These findings would be useful in understanding the evolution of transmitting tract in general and knowledge of pollen-pistil interaction of the species in particular.

## Introduction

A conspecific pollen–pistil interaction encompassing pollen germination on a stigma, pollen tube growth and guidance through the style followed by successful fertilization constitutes the progamic phase in angiosperms (Heslop-Harrison 1975*a*, *b*; Linskens 1986). The functional attributes of the phase, such as duration, male gametophyte competition and regulation of the number of pollen tubes (selection/screening), are controlled by structural modifications that have accompanied the closure of carpels (angiospermy) in flowering plants (Bell 1995; Herrero and Hormaza 1996; Sage *et al.* 2009; Williams *et al.* 2010). Among these modifications, specialization of an elaborate receptive surface into a terminal stigma and the internalized pollen tube transmitting tract (internal compitum) are two of the conspicuous developmental events (Endress and Igersheim 2000; Williams 2009; Endress 2011). These processes have apparently followed different evolutionary lines (Endress 1982; Scutt *et al.* 2006) and may significantly reflect the habitat conditions or the pollination syndromes (biotic and/or abiotic) which proved effective in maximizing fitness in a species (Wang *et al.* 2011).

The pollen tube path in a majority of plants has evolved from an extragynoecial compitum to an internalized one through syncarpy, which has offered greater advantages by providing favourable conditions and the necessary nutrition for a prolonged, lengthier and even distribution of tube growth inside the pistil (Labarca and Loewus 1973; Herrero and Dickinson 1979; Herrero and Hormaza 1996). In spite of these advantages, there have been evolutionary reversals to apocarpy and/or unicarpellate condition (Endress 2011; Prychid *et al.* 2011; Wang *et al.* 2011). Nearly 10 % of the angiosperms have apocarpous gynoecia and 10 % are unicarpellate (Endress 1994, 2001). Apocarpy has greater preponderance in basal eudicots (Endress and Igersheim 2000; Endress and Doyle 2009). In higher eudicots and monocots, apocarpy is thought to be secondarily derived (Endress *et al.* 1983). As the mechanism of angiospermy has largely evolved independently from syncarpy (carpel fusion) (Williams *et al.* 2010), studies on core eudicots with unicarpellate or apocarpous gynoecia may provide fresh insights into the evolution and modularity in the structure and function of the progamic phase.

Elaeagnaceae, a core eudicot family nested in Rosales, exhibits apocarpous/unicarpellate condition and shares this feature with its sister family Barbeyaceae (Dickinson and Sweitzer 1970; Thulin *et al.* 1998). In its related families Rhamnaceae and Dirachmaceae, syncarpy is a prevalent condition. In *Colletia spinosissima* (Rhamnaceae), an extragynoecial compitum is present in the form of stigmatic exudate (Medan and Basilio 2001). Elaeagnaceae is represented by three genera: *Hippophae*, *Elaeagnus* and *Shepherdia*. The structural details of the gynoecia and information on the progamic phase in these species are not available. Among these, *Hippophae rhamnoides* (Himalayan sea buckthorn) is an Old World species distributed from Europe to China across the Himalaya (Mabberley 2008). It is a small to medium-size dioecious shrub and is largely exploited for its berries, known as Leh berry in India, from the wild. There are attempts to bring the species under cultivation and breeding programmes for sustainable utilization. Knowledge of the progamic phase including pollen–pistil interaction would be useful for these endeavours. In the present work on *H. rhamnoides*, we provide a detailed account on the structural and functional aspects of its gynoecium, and demonstrate that the species exhibits several plesiomorphic features in the gynoecium and a partly internalized pollen tube path.

## Methods

### Study species

In India, *H. rhamnoides* grows naturally between 3000 and 5000 m above sea level in the State of Jammu and Kashmir, Himachal Pradesh, Uttarakhand and Sikkim (Raina *et al.* 2012). The study material was collected during two flowering seasons (April/May, 2010 and 2011), from two natural populations in Leh (Jammu & Kashmir): located along the Indus River at Choglamsar (34°05.236′N, 077.36.090′E) and at Sindhu Darshan (34°05.269′N, 077.36.687′E). The climate during the flowering period is generally windy and the mean ambient temperature ranges between −12 and 14 °C.

The female flowers of sea buckthorn are borne in condensed axillary and terminal racemes. Each female flower is pedicellate and the gynoecium is enveloped in two partly fused perianth lobes. Anthesis in female flowers is marked by the emergence of a yellowish-green stigma from the perianth lobes, between 09:00 and 12:00 h **[see Supporting Information]**.

### Gynoecium architecture

Freshly opened female flowers (*n* = 100) were dissected from randomly collected inflorescences and the morphological details of flowers were studied with the aid of a stereo-zoom microscope (Magnüs MS 24, Olympus, India). The morphometric details were recorded with the help of a calibrated ocular micrometer (Erma, Tokyo).

For anatomical details, the pollinated and unpollinated gynoecia were fixed in Karnovsky's fixative for 4 h, rinsed with freshly prepared sodium cacodylate buffer (0.2 M) twice, dehydrated in an ethanol series, and then infiltrated and embedded in a glycol methacrylate monomer mixture (Merck). The sections (2, 4 and 5 µm) were obtained using glass knives on a rotary microtome (AO Spencer, USA), stained with toluidine blue O′ (Sigma, pH 4.4; Feder and O'Brien 1968) and viewed under a photomicroscope (Primo Star, Carl Zeiss, Germany). Additionally, some sections were stained with oil red (EX1-359 or EX2-518) to localize the lipids (Lillie and Fuller 1976), auramine-O (0.01 % in 0.05 M Tris/HCl buffer, pH 7.2, EX-460 nm; EM-550 nm) for cuticle and lipids (Heslop-Harrison and Shivanna 1977), 8-ANS (0.001 % 8-anilino-1-naphthalene sulfonic acid, EX-380 nm; EM-470 nm) for proteins (Mattsson *et al.* 1974) and PAS (periodic acid Schiff's reagent) for polysaccharides (McGukin and Mackenzie 1958).

### The pollen tube path and progamic phase

The pollen tube path was established by localizing the tubes from manually pollinated and open-pollinated gynoecia. For manual pollinations, the most receptive stage of stigma was ascertained through the peroxidases test (Shivanna and Rangaswamy 1992). The pollinated gynoecia (*n* = 450) were fixed in a lactic acid–ethanol (70 %) solution (1 : 3 v/v) at intervals of 12, 20, 24, 30, 36, 42, 48, 56, 60, 70 and 72 h after pollination (hap), for 4 h at 4 °C, rinsed with 70 % ethanol and then stored in the same solution. The temporal details of tube growth in the pollinated gynoecia were established by the decolourized aniline blue staining method (Martin 1959). Observations were made under an epifluorescence photomicroscope (Eclipse 80i Nikon, Japan). The mean tube length ± standard deviation, at each time interval, was computed and plotted against the duration at which the gynoecia were chemically fixed. The average growth rate of the pollen tube was computed by dividing the mean length of pollen tubes (*n* = 20 pollinated gynoecia) by 48 h (i.e. 72 h minus 24 h), because the grains took nearly 24 h to show significant germination. Pollen grains with a tube nearly equal to the diameter of pollen grains were considered germinated.

### Electron microscopy

For scanning electron microscopy, gynoecia were fixed in Karnovsky's fixative (4 h, 4 °C), dehydrated using a cold acetone series (from 10 to 100 %), dried to the critical point (E-3000, Quorum Technologies, UK), sputter-coated with gold–palladium (JFC-1600 Autofine Coater, Jeol, Japan) and observed under a scanning electron microscope (JSM-6610LV-Jeol, Japan).

The ultrastructural details of the cells lining the pollen tube path were studied by transmission electron microscopy. For this, the gynoecia were fixed in Karnovsky's fixative for 4 h at 4 °C, and washed twice with sodium phosphate buffer (0.2 M). Post-fixation was done in osmium tetroxide (1 %) prepared in the buffer, for 2 h at 4 °C, dehydrated through ascending cold acetone series and embedding was done in a CY212 araldite–resin mixture. Ultrathin sections (60–90 nm) were obtained using an ultracut rotary microtome (E-Reichert, Germany), collected on carbon-coated copper grids and stained with alcoholic uranyl acetate for 10 min followed by staining with lead acetate for 8 min (Reynolds 1963). Observations were made under a transmission electron microscope (Morgagni 268D, FEI Electron Optics).

## Results

### Structural organization of the gynoecium

**Figure 1.**
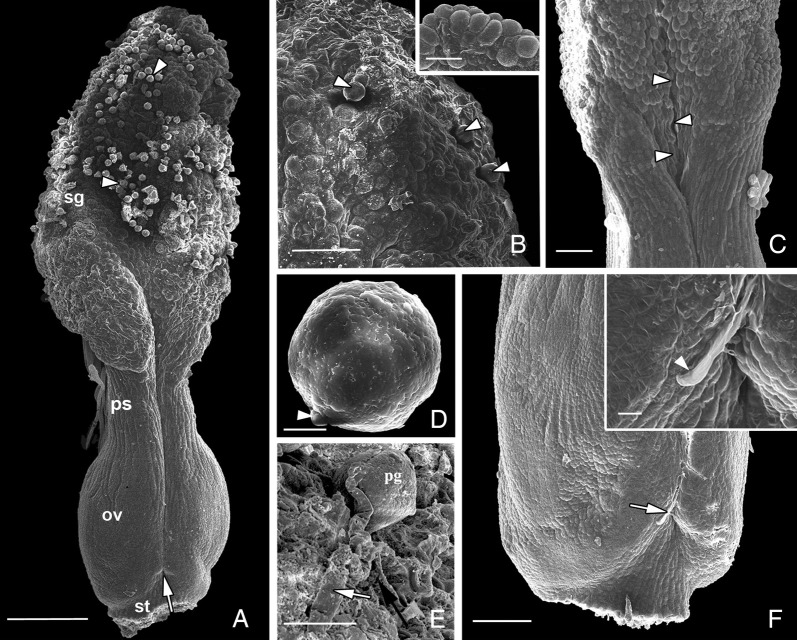
Scanning electron micrographs illustrating the gynoecium architecture, pollen morphology and events of progamic phase in *H. rhamnoides.* (A) Ventral side of manually pollinated gynoecium with stipe, showing abundant pollen (arrowheads) on stigma, conduplication along the pseudostyle and ovary region which ends up in a conspicuous pore (ventral pore, arrow). (B) Part of an open-pollinated stigma showing deposition of pollen grains (arrowheads). Note that pollination causes deposition of exudates/ECM on the stigma surface due to cuticle discontinuities. Inset: Part of the unpollinated stigma at anthesis. Absence of ECM or exudates clearly indicates that the stigma is of dry type. (C) Close-up of the stigma–pseudostyle interface, and pseudostyle. Arrowheads point to the ventral slit. (D) A magnified pollen grain exhibiting smooth exine and germ pore (arrowhead). (E) Germinated pollen (30 h after pollination) showing initial phase of pollen tube growth on the stigmatic surface (arrowhead) before penetration into stigmatic tissue (arrow). (F) Ovary of a pollinated gynoecium (60 h after pollination) showing the pollen tube (arrow) near the ventral pore. The inset shows the magnified view of the pollen tube tip (arrowhead) near the pore. Abbreviations: ECM, extracellular matrix; ov, ovary; pg, pollen grain; ps, pseudostyle; sg, stigma; st, stipe. Scale bars: A = 200 μm; B = 50 μm (inset = 50 μm); C = 50 μm; D = 5 μm; E = 20 μm; F = 100 μm (inset = 10 μm).

**Figure 2.**
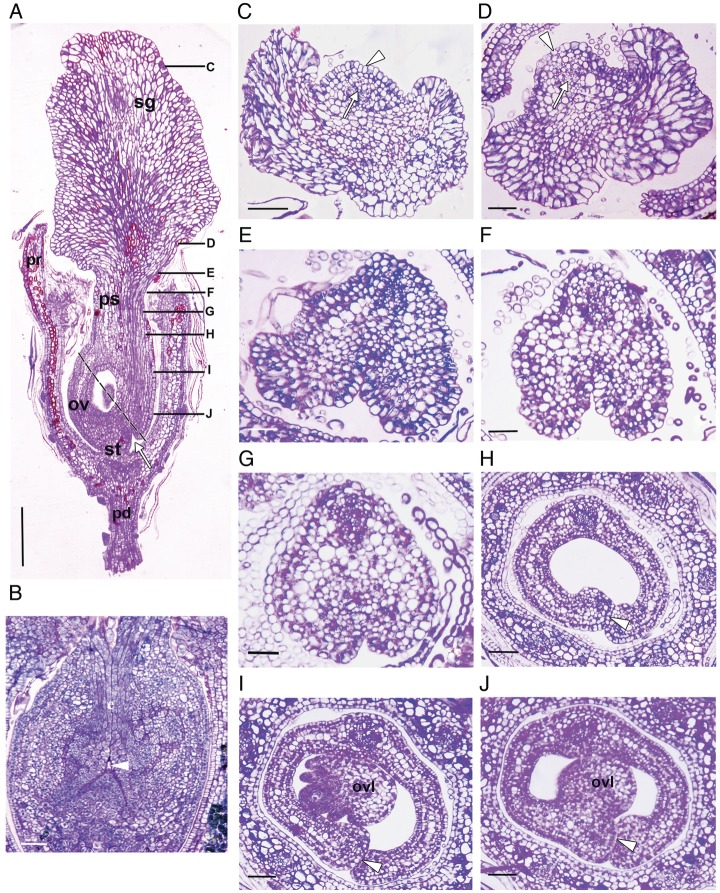
Anatomical details of the female flower showing the conduplicate gynoecium. (A) Longitudinal section of a young female flower showing different parts of the gynoecium. Note that the gynoecium is shortly stipitate. The black broken line shows the plane of section of (B) of the gynoecium. (B) An oblique longitudinal section of the gynoecium from the proximal region of the gynoecium showing the ovarian pore (arrowhead) through which the pollen tube makes its entry to the ovarian locule. (C–J) Transverse sections of a young gynoecium. The corresponding regions of sections are marked in (A). The lower side of the sections represents the ventral side. (C–E) Dorsal surface of the gynoecium at the stigma region, marked by a thick cuticle (arrowhead). A solitary dorsal vascular supply is noticeable (arrow). (F and G) Pseudostyle (ps) region. A transmitting tissue (compitum) is absent. (H) Ovarian locule. Note the continuity of the locular epidermis (arrowhead) with the periphery and at the meeting point of the carpellary margins. (I) The mid-ovarian region, showing a bitegmic ovule and a developing female gametophyte. (J) The ovarian region near the pore; continuous epidermis (arrowhead) still encircles the region and shows that ovule is borne out from the submarginal position of the carpel wall. Abbreviations: ov, ovary; ovl, ovule; pe, pedicel of flower; pr, perianth; ps, pseudostyle; sg, stigma; st, stipe. Scale bars: A = 500 μm; B = 100 μm; C–E = 50 μm; F = 50 μm; G = 20 μm; H = 20 μm; I = 50 μm; J = 50 μm.

The gynoecium of sea buckthorn is unicarpellate and the carpel exhibits a distinct dorsiventral symmetry. It is shortly stipitate and measures 3.4 ± 0.4 mm in length (*n* = 30) (Fig. 1A). The ventral side is marked by an apical and prominently fanned out stigma. There is a continuous groove (ventral slit) that runs downwards, half-way from the stigma to the sub-basal region of a dorsally protruded ovary (Figs 1A and 2A).

**Figure 3.**
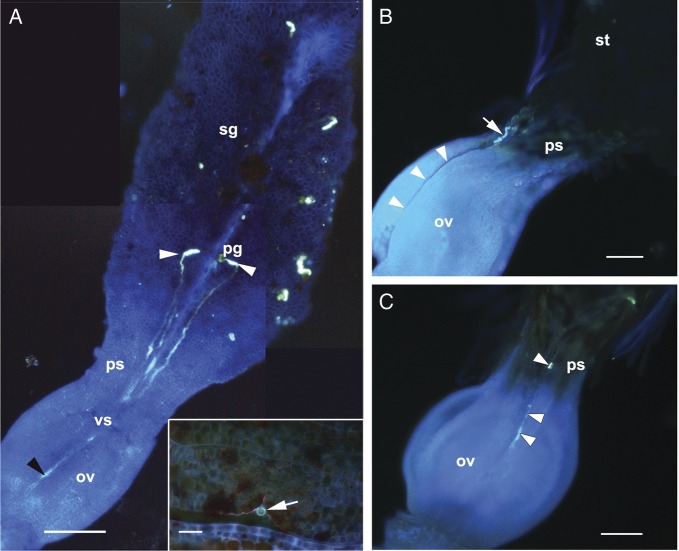
Epifluorescence photomicrographs of pollinated gynoecium. (A) A gynoecium 72 h after pollination showing the pollen tube along the ventral slit. Note that the tubes are directed towards the ventral slit in the pseudostyle and ovary region. The black arrowhead shows the point of entry of the pollen tube into the ovarian locule. The inset shows the pollinated gynoecium in cross-section at the ovarian region with a fluorescing pollen tube (arrow) near the ovarian pore. (B and C) A gynoecium 48 h after pollination showing a pollen tube at the stigma–pseudostyle (arrow) interface and its growth towards the slit (arrowheads, B) and along the conduplicate zone (slit, arrowheads) of the ovarian region (C). Abbreviations: ov, ovary; pg, pollen grain; ps, pseudostyle; pt, pollen tube; sg, stigma; vg, ventral slit. Scale bars: A, B and C = 100 μm; A (inset) = 25 μm.

A freshly emerged yellowish-green stigma is the most conspicuous part of the gynoecium (2.0 ± 0.1 mm in length, n = 30), which becomes exposed to air, like a baseball glove. It is dry and of non-papillate type (Heslop-Harrison and Shivanna 1977) with an undulated (scorbiculate) surface (Figs 1B, 2C and D).

The stigma is subtended by a short constricted region (0.3 ± 0.1 mm in length), termed here as the pseudostyle (Figs 1A and 2A). The internalized transmitting tissue (compitum)-like organization is absent in this zone (Figs 2F and G). The margins of the stigma taper and merge downwards in this region to form a stigma–pseudostyle junction (Figs 1A, C and 2E). The corresponding dorsal surface of the pseudostyle is covered with dendroid trichomes (Figs 1A and 2E). The ventral slit becomes conspicuous in the pseudostyle region due to involution of the carpel margins (Fig 1A and C).

**Figure 4.**
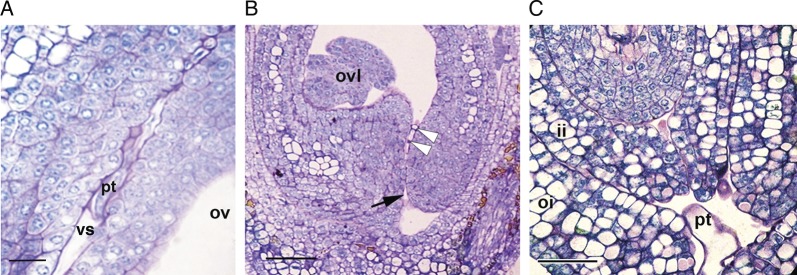
Pollen tube path near the ovule. (A) The pseudostyle–ovary interface with a pollen tube growing along the ventral slit. (B) A pollen tube entering (arrowheads) through the ovarian pore. The black arrow shows the external opening of the pore. (C) Longitudinal section of the ovule showing pollen tube entry into the micropyle. Abbreviations: ii, inner integument; oi, outer integument; ov, ovary; ovl, ovule; pt, pollen tube; vs, ventral slit. Scale bars = 50 μm.

The ovary (0.8 ± 0.1 mm in length) is superior, unilocular with a single bitegmic, anatropous and crassinucellate ovule (Fig. 2A and I). The ovarian region is distinctly conduplicated as is evident by a ventral slit, which eventually leads to an ovarian pore near the base of the ovary (Figs 1A, F, 2B and 3B). The ovarian pore makes a continuum between the pollen tube path and the locule of the ovary (Figs 2H–J and 4B). The ovule develops from the submarginal position on the ventral carpellar wall of the locule (Fig. 2J). The micropyle invariably faces away from the ovarian pore (Fig. 2I).

### The progamic phase and pollen tube path

**Figure 5.**
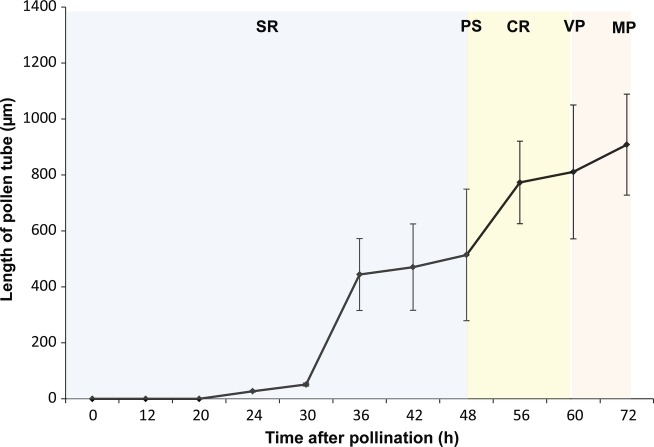
Temporal details of the pollen tube growth of *H. rhamnoides* at different intervals of time and locations in the pollinated gynoecium. The data represent the average tube length (±standard deviation). Abbreviations: CR, conduplicate region; PS, pseudostyle; SR, stigmatic region; VP, ventral pore; MP, micropyle.

The pollen grains measured 26.47 ± 3.27 μm in diameter (*n* = 100 pollen grains, 10 flowers) and are shed from anthers at the two-celled stage **[see Supporting Information]**; the exine is smooth (Fig. 1D). On average, the pollen grains took ∼20 h to produce a small protuberance from the germ pore. After 24 h of pollination, 81.88 ± 14 % of the pollen showed tube emergence nearly equal to the mean diameter of pollen grain (27.05 ± 3.01 μm, *n* = 18 gynoecia). Thirty hours after pollination, the tubes attained a length of 51.0 ± 5.41 μm (*n* = 30 gynoecia) and penetrated into the subdermal tissue (Figs 1E and 5).

**Figure 6.**
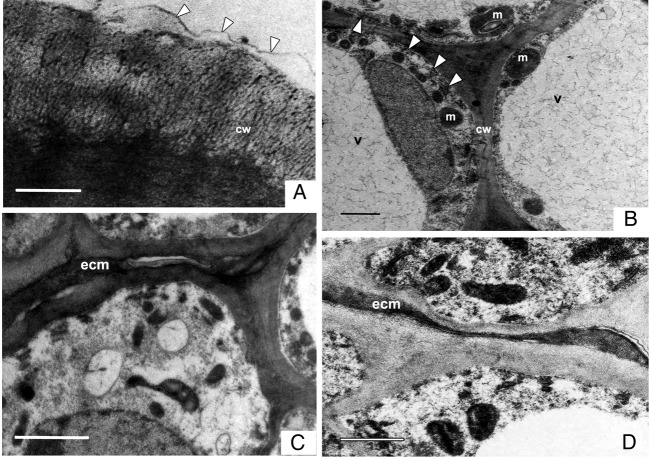
Transmission electron micrographs of different regions of the gynoecium. (A) A part of the stigmatic cell with an undulating cuticle (arrowheads) forming irregular bulges over the cell wall. (B) Large, vacuolated cells of stigma with intercellular spaces. The stigmatic cells contain numerous rough endoplasmic reticulae (arrowheads) and mitochondria along the plasma membrane, indicating their secretory nature. (C) Extracellular matrix accumulated in the interstitial spaces of the hypodermal region in the pseudostyle along the site of conduplication. (D) Sparsely localized ECM between the epidermal cells lining the pore. Abbreviations: cw, thick cell wall; ecm, extracellular matrix; m, mitochondria; n, nucleus; rer, rough endoplasmic reticulae; v, vacuole. Scale bars: A = 0.5 μm; B and C = 2 μm; D = 1 μm.

**Figure 7.**
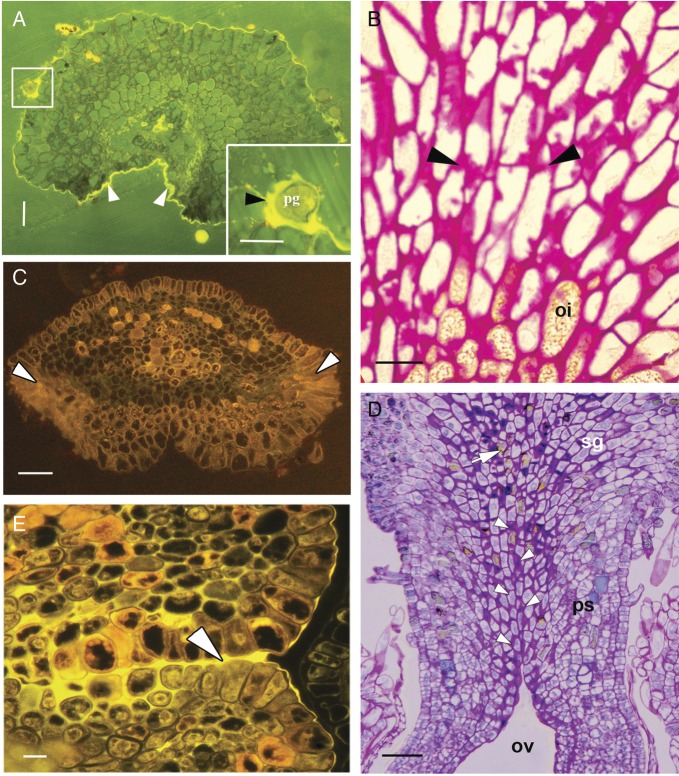
Histochemistry of the pollen tube path in *H. rhamnoides.* (A) Pollinated stigma, stained with auramine-O, showing the presence of cuticle. The cuticle is thicker over the dorsal surface (arrowheads). The inset shows the pollen grain submerged in the ECM deposited over the stigmatic surface, after pollination due to the development of discontinuities in the cuticle layer. (B) Stigmatic cells in the longitudinal section showing accumulation of the ECM, rich in carbohydrate (PAS positive), within the intercellular spaces (arrowheads) and the oil in some of the cells. (C) Stigma of the pollinated gynoecium, stained with 8-ANS, showing deposition of proteins towards the ventral surface (arrowheads). The ventral surface is at the top. (D) Longitudinal section of a part of the stigma (toluidine blue O′ stained), pseudostyle and upper region of the ovary showing ECM accumulation in the intercellular spaces of the stigmatic cells and along the ventral groove (arrowheads). The arrow shows one of the oil-containing cells. (E) The pseudostyle region stained with oil red, showing the presence of lipids in the ECM (arrowhead), accumulated beneath the ventral slit. Abbreviations: ECM, extracellular matrix; oi, oil. Scale bars: A = 50 μm; inset = 20 μm; B = 10 μm; C = 50 μm; D = 50 μm; E = 50 μm.

The stigma attains receptivity soon after its emergence from the perianth and reaches its peak once it outgrows the subtending bract. Before pollination, the stigma is covered with a continuous cuticle, which is thicker over the median dorsal and non-receptive surface. After pollination, the cuticle lining the ventral surface becomes discontinuous (Figs 6A and 7A). Five or six layers of the subdermal cells of stigma are large, vacuolated and possess intercellular spaces. These interstitial spaces accumulate a predominantly polysaccharidic and proteinaceous extracellular matrix (ECM) (Figs 6B, 7B and C). The secretory nature of these cells was evident from the presence of numerous mitochondria and endoplasmic reticulae along the plasma membrane (Fig. 6B). The marginal cells of stigma showed accumulation of oils and phenolics (Fig. 7B, D and E).

Tubes grew through the intercellular spaces of the substigmatic tissue, before reaching the stigma–pseudostyle junction, 48 hap (Figs 3A, B and 5). Interestingly, after a subdermal phase of growth, the tube(s) emerge near the junction and grow over the epidermal surface of the slit before making their entry into the locule through the ovarian pore (Figs 3, 4A and B).

Pollen tube growth along the ventral slit is supported by ECM secreted from the secretory activity of juxtaposed cells. In the pseudostyle region, the cells lining the slit are smaller, globular and compactly arranged whereas the hypodermal region possess interstitial spaces. These spaces accumulate osmiophilic and protein-rich ECM (Figs 6C, 7D and E); carbohydrates are sparsely distributed.

The pollen tube reaches the pore (Figs 1F, 3C and 4B) by 60 hap (*n* = 20 gynoecia) and enters the micropyle (Fig. 4C) by ∼72 hap (*n* = 15 gynoecia). The ventral slit in the ovarian region and the ovarian pore is lined by a continuous layer of compactly arranged and thick-walled epidermis (Fig. 2H–J). A sparse osmiophilic ECM could be localized all along the ventral slit of the ovarian region and in the intervening space of the ovarian pore (Fig. 6D).

In nearly 90 % of the instances (*n* = 30), only one pollen tube traversed through the pseudostyle, and in the remaining 10 %, two pollen tubes were observed (Fig. 3A). In the latter case, however, invariably only one pollen tube could make its entry through the ovarian pore (Fig. 3C) and reached the micropyle. The mean pollen tube length in the pollinated gynoecia after 72 h was 908.13 ± 180.43 µm (range: 600–1300 µm, Fig. 5). Thus, the pollen tube growth rate from the time of pollen germination to ovule penetration was 18.75 µm h^−1^.

## Discussion

This is the first comprehensive account of the structural and functional aspects of the progamic phase in any taxa of Elaeagnaceae. Many character states in *H. rhamnoides* are plesiomorphic, as they have preponderance in basal angiosperms and the basal eudicots, and provide new insight into the pollen tube path.

### Structural organization of the gynoecium

The gynoecial features in sea buckthorn, such as conduplication, sparse exudation of ECM along the site of angiospermy and incomplete fusion of carpel margins, together strongly suggest that angiospermy in sea buckthorn is contributed mainly by secretion and to some extent by the closely appressed carpel margins (Lloyd and Wells 1992; Endress and Igersheim 2000). The condition is close to type 2 angiospermy, where carpel closure is achieved by secretion and postgenital fusion (Endress and Igersheim 2000). The intervening constricted region between the stigma and ovary lacks the usual specialized transmitting tissue (compitum), and hence is termed a pseudostyle rather than a style (Bailey and Swamy 1951).

More than half of the ventral (adaxial) surface is receptive (stigma). This is in contrast to some extant basal angiosperms with style-less, conduplicated carpels where the receptive secretory/stigmatic hairs extend to the inner surface of the carpel and pollen grains are received on two independent papillate margins known as stigmatic crests (Bailey and Swamy 1951; Endress 2005). During the evolutionary modification of the carpel, deformation of either the abaxial or adaxial surface is instrumental in displacing the stigma from a stigmatic crest to the terminal region of the style (Bailey and Swamy 1951). In *H. rhamnoides*, adaxial deformation seems to have developmentally organized the stigma.

An incomplete connation of carpel margins in sea buckthorn is also evident from the continuation of their epidermis into the ovarian locule, which leaves a ventral pore in the lower end of the slit. In taxa such as *Kadsura*, *Schisandra* (Schisandraceae) and *Sagittaria* (Alismataceae), the ventral pore may serve as the exit as well the entry point of pollen tubes, in a continuum of extragynoecial compitum (Lyew *et al.* 2007). In *H. rhamnoides*, the ventral pore is the only route taken by the pollen tubes to gain access to the ovule.

The formation of a hunchback-like and dorsally protruded ovary in sea buckthorn is largely a consequence of the syntropous orientation of anatropous ovule, which is most likely accompanied by conduplication to accommodate the more or less basally positioned ovule. This causes the micropyle of the ovule to face away from the ventral pore. A similar condition is seen where an ovule borne on the submarginal position in free carpels is curved away from the margins in the direction of involution (Endress 1994). The dorsal bulge in the ovary around the ovule is considered an evolutionary specialization during carpel development, as in some taxa of Monimiaceae with a weakly peltate carpel and a dorsal bulge (Endress 2011).

### The progamic phase and pollen tube path

Sea buckthorn is a wind-pollinated plant and inhabits ecological conditions suitable for anemophily. The floral contrivances such as dry stigma, copious production of dry, starchy pollen with smooth exine and their morphometric range are similar to a majority of anemophilous taxa (Culley *et al.* 2002). Further, a large stigma in *H. rhamnoides* is also an obvious adaptive attribute to capture the airborne pollen grains.

During the functional phase of a dry stigma, the cuticle–pellicle layer is usually complete (Heslop-Harrison and Shivanna 1977). The cuticle layer develops discontinuities after pollination, which is accompanied by accumulation of secretion products beneath the cuticle–pellicle layer. This build-up of stigmatic ECM results in the formation of irregular bulges over the stigmatic surface, as seen in other systems with dry stigma (Shivanna and Johri 1985). The stigmatic ECM available through the cuticular discontinuities possibly facilitates adhesion and hydration of airborne pollen grains of species with dry stigma (Elleman *et al.* 1992).

Except for the fact that the species lacks an extragynoecial compitum by virtue of having a solitary and unicarpellate gynoecium, the partly hidden pollen tube growth pattern is similar to that reported in Winteraceae (Frame 2003), Schisandraceae (Lyew *et al.* 2007) and the other basal angiosperms (Endress and Igersheim 2000; Koehl 2002; Bernhardt *et al.* 2003; Thien *et al.* 2003; Lora *et al.* 2010). Enfolding of the carpel margins in the pseudostyle offers protection to the growing pollen tube (Lyew *et al.* 2007) and to a minor amount of ECM present over the secretory slit. The role of the pseudostyle is more or less similar to that of the usual compitum in confining the pollen tube growth along the secretory path and regulating the number of pollen tubes (Erbar 2003). In *Annona cherimola* and *Amborella trichopoda*, the stigma–style interface has been described as the site where male gametophyte selection and reduction in the number of pollen tubes occur; and only one or two pollen tubes enter the stylar canal (Williams 2009; Lora *et al.* 2010). In *H. rhamnoides*, male gametophyte competition is possibly confined to the stigmatic region and those with greater vigour nearly reach the pseudostyle. However, further down, the most vigorous one is able to grow beyond the stigma–pseudostyle interface due to spatial constraints (Erbar 2003) and thus in this context the interface appears to serve as the site of regulation of tube number.

The pollen tube growth rate is species specific and may be influenced by the length of the pollen tube path and ambient temperature regimen (Williams 2008, 2009; Steinacher and Wagner 2012). The average growth rate of the pollen tube in *H. rhamnoides* (18.75 µm h^−1^) is comparable to that of gymnosperms such as *Ephedra distachya* (14 µm h^−1^) and *E. trifurca* (6–19 µm h^−1^) (Williams 2009). In *H. rhamnoides*, the slow metabolic growth due to sub-zero temperature could be the cause of a slower tube growth rate.

## Conclusions

In the evolutionary history of flowering plants, several ancestral reproductive features in the relatively advanced taxa are believed to be a consequence of reversals (Doyle and Endress 2000; Endress and Doyle 2009; Endress 2011). The presence of many plesiomorphic traits in the gynoecia of derived lineages suggests homoplasic apomorphy either by reversion or secondary derivation (Endress 1982), which is most likely influenced by the fitness requirements under the prevailing ecological conditions of a plant species (Armbruster *et al.* 2002). Additionally, many features such as open habitats, dioecy, simple and small flowers, uniovulate condition and wind pollination have been shown to exhibit correlated evolution (Friedman and Barrett 2008). The occurrence of conduplication, an incomplete connation of the carpellar margins resulting in a ventral pore and a partly internal progamic phase represent a peculiar combination of gynoecial features in *H. rhamnoides*, a core eudicot nested within Rosales (APG III 2009). It is likely that, together, these features are the derived ones that have accompanied modification of the gynoecium of sexually diverging individuals in a dioecious system.

## Sources of Funding

This work was financially supported by the University Grants Commission, India [F. No. 37-405/2009(SR)] and the Department of Biotechnology (DBT) (BT/PR10800/NDB/51/172/2008), Government of India.

## Contributions by the Authors

Y.M. and R.T. made equal contributions in conducting the research. R.T. and S.G. were involved in planning the research and in writing the manuscript. S.N.R. was involved in the planning and supervision of the research work.

## Conflict of Interest Statement

None declared.

## Supporting Information

The following Supporting information is available in the on line version of this article.

**Fig. 1.** (A) A flowering branch of female plant. Arrows shows the freshly emerged stigma in inflorescences. (B) Scanning electron micrograph of mature female flower (lateral view) at anthesis. Note the presence of trichomes over the perianth and the bract. (C) Confocal laser scanning micrograph (model: LSM5 Pascal, Carl Zeiss, Germany) of fresh pollen grains, stained with propidium iodide. Arrows indicate two nuclei in a pollen grain. Abbreviations: pr, perianth; br, bract; st, stigma. Scale bars: A = 1 mm, B = 200 µm, C = 25 µm.

Supplementary Data
