# Supplementary material for: Structural organization of the gynoecium and pollen tube path in Himalayan sea buckthorn, Hippophae rhamnoides (Elaeagnaceae)
Source: AoB Plants. 2013 Feb 27;5:plt015. doi: 10.1093/aobpla/plt015 (PMC4130438; doi:10.1093/aobpla/plt015)
Supplement: Supplementary Data [file supp_5_plt015_index.html]

Structural organization of the gynoecium and pollen tube path in Himalayan sea buckthorn, Hippophae rhamnoides (Elaeagnaceae) — Supplementary Data 

# Structural organization of the gynoecium and pollen tube path in Himalayan sea buckthorn, *Hippophae rhamnoides* (Elaeagnaceae)

## Supplementary Data

Supplementary Data

**Files in this Data Supplement:**

- Supplementary Data - Doc file
