# Supplementary material for: Structural organization of the gynoecium and pollen tube path in Himalayan sea buckthorn, Hippophae rhamnoides (Elaeagnaceae)
Source: AoB Plants. 2013 Feb 27;5:plt015. doi: 10.1093/aobpla/plt015 (PMC4130438; doi:10.1093/aobpla/plt015)
Supplement: Supplementary Data [file supp_plt015_plt015supp.doc]

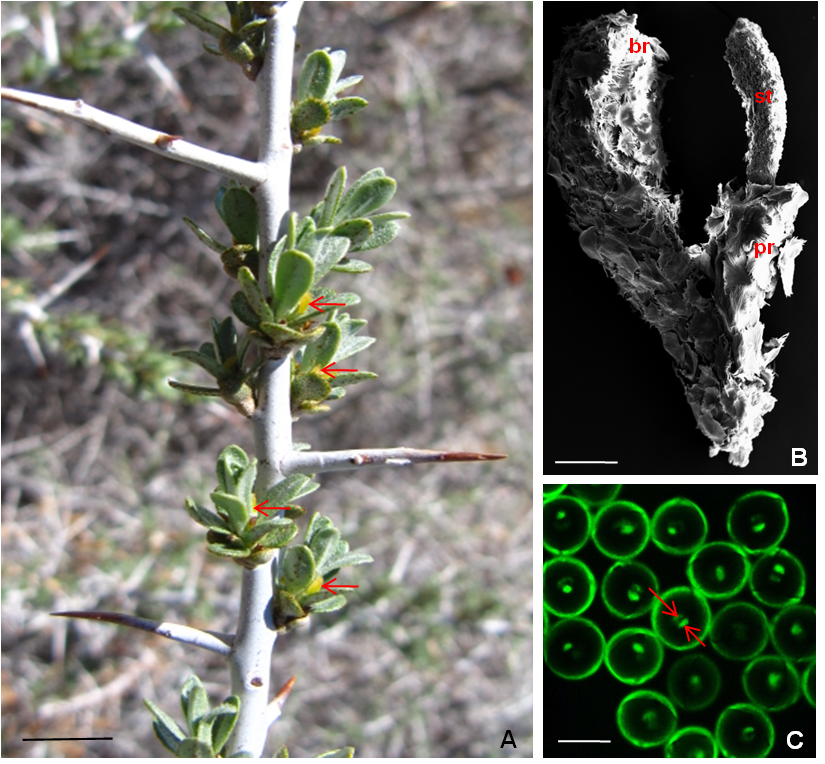


**Supplementary Figure 1:**

(A) A flowering branch of female plant. Arrows shows the freshly emerged stigma in inflorescences.

(B) Scanning electron micrograph of mature female flower (lateral view) at anthesis. Note the presence of trichomes over the perianth and the bract.

(C) Confocal laser scanning micrograph (model: LSM5 Pascal, Carl Zeiss, Germany) of fresh pollen grains, stained with propidium iodide. Arrows indicate two nuclei in a pollen grain.

Abbreviations: pr, perianth; br, bract; st, stigma.

Scale bars: A = 1mm, B = 200 μm, C = 25 μm.
